# Impact of dual mTORC1/2 mTOR kinase inhibitor AZD8055 on acquired endocrine resistance in breast cancer *in vitro*

**DOI:** 10.1186/bcr3604

**Published:** 2014-01-23

**Authors:** Nicola J Jordan, Carol M Dutkowski, Denise Barrow, Huw J Mottram, Iain R Hutcheson, Robert I Nicholson, Sylvie M Guichard, Julia MW Gee

**Affiliations:** 1 Breast Cancer (Molecular Pharmacology) Group, Cardiff School of Pharmacy and Pharmaceutical Sciences, Cardiff University, Redwood Building, King Edward VII Avenue, Cardiff CF10 3NB, UK; 2 Department of Pharmacology, Therapeutics & Toxicology, Cardiff University School of Medicine, UHW Main Building, Heath Park, Cardiff CF14 4XN, UK; 3 Oncology, AstraZeneca, Alderley Park, Macclesfield, Cheshire SK10 4TG, UK; 4 CUPUCI, School of Medicine, Cardiff University, Henry Wellcome Building, Heath Park, Cardiff CF14 4XN, UK

## Abstract

**Introduction:**

Upregulation of PI3K/Akt/mTOR signalling in endocrine-resistant breast cancer (BC) has identified mTOR as an attractive target alongside anti-hormones to control resistance. RAD001 (everolimus/Afinitor®), an allosteric mTOR inhibitor, is proving valuable in this setting; however, some patients are inherently refractory or relapse during treatment requiring alternative strategies. Here we evaluate the potential for novel dual mTORC1/2 mTOR kinase inhibitors, exemplified by AZD8055, by comparison with RAD001 in ER + endocrine resistant BC cells.

**Methods:**

*In vitro* models of tamoxifen (TamR) or oestrogen deprivation resistance (MCF7-X) were treated with RAD001 or AZD8055 alone or combined with anti-hormone fulvestrant. Endpoints included growth, cell proliferation (Ki67), viability and migration, with PI3K/AKT/mTOR signalling impact monitored by Western blotting. Potential ER cross-talk was investigated by immunocytochemistry and RT-PCR.

**Results:**

RAD001 was a poor growth inhibitor of MCF7-derived TamR and MCF7-X cells (IC_50_ ≥1 μM), rapidly inhibiting mTORC1 but not mTORC2/AKT signalling. In contrast AZD8055, which rapidly inhibited both mTORC1 and mTORC2/AKT activity, was a highly effective (*P* <0.001) growth inhibitor of TamR (IC_50_ 18 nM) and MCF7-X (IC_50_ 24 nM), and of a further T47D-derived tamoxifen resistant model T47D-tamR (IC_50_ 19 nM). AZD8055 significantly (*P* <0.05) inhibited resistant cell proliferation, increased cell death and reduced migration. Furthermore, dual treatment of TamR or MCF7-X cells with AZD8055 plus fulvestrant provided superior control of resistant growth versus either agent alone (*P* <0.05). Co-treating with AZD8055 alongside tamoxifen (*P* <0.01) or oestrogen deprivation (*P* <0.05) also effectively inhibited endocrine responsive MCF-7 cells. Although AZD8055 inhibited oestrogen receptor (ER) ser167 phosphorylation in TamR and MCF7-X, it had no effect on ER ser118 activity or expression of several ER-regulated genes, suggesting the mTOR kinase inhibitor impact was largely ER-independent. The capacity of AZD8055 for ER-independent activity was further evidenced by growth inhibition (IC_50_18 and 20 nM) of two acquired fulvestrant resistant models lacking ER.

**Conclusions:**

This is the first report demonstrating dual mTORC1/2 mTOR kinase inhibitors have potential to control acquired endocrine resistant BC, even under conditions where everolimus fails. Such inhibitors may prove of particular benefit when used alongside anti-hormonal treatment as second-line therapy in endocrine resistant disease, and also potentially alongside anti-hormones during the earlier endocrine responsive phase to hinder development of resistance.

## Introduction

Growth of the majority of breast cancers is stimulated by oestrogen and this oestrogen receptor (ER) signalling can be successfully blocked by anti-hormonal treatments, including aromatase inhibitors or the oestrogen receptor antagonists, tamoxifen or fulvestrant. Anti-hormone treatment is effective in a high proportion of initially responsive patients but subsequently a significant number acquire resistance with resulting poorer survival rates [1]. Consequently, there is an urgent need for treatments for breast cancer that improve responses to prevent or delay endocrine resistance. In an attempt to overcome endocrine resistance, studies have focussed on developing novel agents that can reverse resistance by targeting growth factor signalling pathways. Endocrine resistant cells can be highly dependent on the use of activated growth factor signalling pathways including epidermal growth factor receptor (EGFR) and human epidermal growth factor receptor-2 (HER2) [2]. The phosphatidylinositol 3-kinase (PI3K)/Akt/mammalian target of rapamycin (mTOR) signalling network is also often prominent in endocrine resistant breast cancer, extending to tamoxifen resistant and oestrogen deprivation resistant MCF-7-derived cell lines [3-5]. In patients with invasive breast cancer, increased activation of this pathway is associated with poor prognosis [6] and, thus, mTOR has recently been recognised as an important drug target for breast cancer therapy [7].

mTOR is a highly conserved serine/threonine protein kinase that belongs to the PI3K- related family and serves as a central regulator of cell metabolism, growth, proliferation and survival [8]. Extensive knowledge about the function of this protein has come from the experimental use of the natural bacterial antibiotic rapamycin, which inhibits the activity of mTOR. mTOR consists of two separate multi-protein complexes, mTORC1 and mTORC2, that are both activated by growth factor stimulation. The mTORC1 complex is rapamycin sensitive; rapamycin binds the FK506-binding protein (FKBP-12) which binds to and causes allosteric inhibition of the signalling complex mTORC1 [9] which contains mTOR, the regulatory associated protein (RAPTOR), mLST8, PRAS40 and DEPTOR proteins. mTORC1 positively regulates protein translation and synthesis via its main substrates, p70 ribosomal S6 kinase (p70S6K) and the eukaryotic initiation factor 4E binding protein-1 (4E-BP1). Upon phosphorylation, 4E-BP1 dissociates from the mRNA cap-binding protein eIF4E and allows it to interact with eIF4G to form a translation initiation complex [10]. In the less well-defined rapamycin-insensitive mTORC2 complex [11], mTOR is associated with the rapamycin insensitive companion (RICTOR), LST8, mSIN1, PROCTOR and DEPTOR and phosphorylation of 4E-BP1 on t37/46 is also considered rapamycin insensitive [12]. The mTORC2 complex is involved in cytoskeletal organisation via paxillin, rho/rac and PKBα, but it also plays a key role in cell proliferation and survival via activation of serum and glucocorticoid protein kinase 1 (SGK1) and direct activation of Akt [8]. However, the characterisation of mTORC1 and mTORC2 as rapamycin-sensitive and insensitive complexes may not always be entirely accurate, as chronic rapamycin treatment has also been reported to inhibit mTORC2 activity by blocking its assembly [8,13].

The mTOR inhibitor rapamycin (sirolimus) has been used clinically as an immunosuppressant drug in transplant medicine [14]. However, it has recently been realised that the increased activity of the mTOR pathway caused by upstream changes in regulators, such as phosphatidylinositol-3 (PI3)-phosphatase (PTEN) and PI3K, also makes mTORC1 an attractive anti-cancer target [15] and a number of rapamycin analogues (rapalogues) have been produced: RAD001 (everolimus, Afinitor®, Novartis), CCI-779 (temsirolimus, Wyeth) and AP23573 (MK-8669) (ridaforolimus ARIAD and Merck pharmaceuticals). The first clinical cancer trials in metastatic breast cancer with temsirolimus as a monotherapy resulted in only partial responses [16,17]. The unexpectedly modest outcomes, with patients acquiring resistance or exhibiting intrinsic resistance to these allosteric mTORC1 inhibitors, may be associated with a paradoxical increase in the activation of Akt and PI3K caused by inhibition of a negative feedback loop from S6 kinase to IRS-1 in response to mTORC1 inhibition by rapalogues [18-21], with the subsequent enhanced Akt activation reportedly being associated with rapalogue monotherapy failure in some patients [22-24].

However, while monotherapy studies with several signal transduction inhibitors, including rapalogues, have shown only modest success in advanced breast cancers, preclinical data indicate that a combination of anti-hormone and signal transduction inhibitors (STI's) can provide significantly greater inhibition than either agent alone [21]. Although breast cancers may become resistant to first-line anti-hormone treatment they often retain an active ER and will still respond to an alternative endocrine agent as a second-line therapy, but longer-term success is more likely to be achieved by also targeting up-regulated growth factor pathways that can be independent from, or interactive with, ER signalling pathways. Pre-clinical data were supportive of the use of current mTOR antagonists alongside endocrine therapy in breast cancer which resulted in a number of clinical trials using such combination therapies [25]. One of the earliest phase 3 trials combined letrozole and temsirolimus and was used on advanced breast cancer but the trial had to be terminated early due to failure to demonstrate any benefit [19]. Later studies have been more successful with very promising results obtained recently in advanced endocrine resistant disease where RAD001 (everolimus) was used in combination with the steroidal aromatase inhibitor exemestane or with tamoxifen in phase 2/3 trials. These have shown significant improvement in progression-free survival from 4.1 months with exemestane alone to 10.6 months with a combination of exemestane and everolimus in the BOLERO 2 trial [26] and an improved time to progression from 4.5 months with tamoxifen alone to 8.6 months with tamoxifen plus everolimus in the TAMRAD GINECO trial [27]. These clinical findings indicated value for the allosteric mTOR inhibitors used alongside tamoxifen or aromatase inhibitors in advanced endocrine resistant tumours and there has been recent USA Food and Drug Administration approval for everolimus in combination with exemestane in ER+/HER2- metastatic breast cancer after non-steroidal aromatase inhibitor failure [28].

Nevertheless, there remains a group of patients who are initially refractory to everolimus/anti-hormone therapy while others relapse at a later point during such treatment [26,27]. It is feasible that these patients may gain more benefit from treatment with alternative mTOR inhibitors that, unlike rapalogues, are not restricted to inhibition of only mTORC1 signalling. It is thus interesting that several types of new mTOR inhibitors are currently under development. The dual mTOR and PI3K inhibitors (SF1126-Semafore, NVP-BEZ235- Novartis, xL765-Exelis-Sanofi and GDC-0980- Roche-Genentech) simultaneously block both the PI3K and mTOR signalling and, therefore, have the theoretical advantage of totally shutting down the PI3K/Akt/mTOR network [14]. These have the possible drawback of association with greater toxicity but in early small clinical trials are reported to induce stable disease or partial response (see [14]). A new variety of mTOR inhibitors has also recently emerged which are ATP-competitive inhibitors that target the mTOR kinase domain and, thus, dually-inhibit activity of both mTORC1 and mTORC2 complexes. This approach should be an alternative way to mitigate the problem of Akt/PI3K activation by negative feedback seen with rapalogues. Preclinical data for two such agents, PP242 and PP30, suggest that along with the additional benefit of mTORC2 inhibition, these drugs can also be more effective than rapamycin at inhibiting mTORC1 activity [14,29,30]. Several pan mTOR (mTORC1/2) dual kinase inhibitors (AZD8055 and its related compound AZD2014 (Astra Zeneca), as well as INK128 (Intellikine) and OSI-027 (OSI Pharmaceuticals)) are currently in phase I/II studies on solid tumours and breast cancer or lymphoma [30-33]. However, the value of such dual mTORC1/2 inhibitory strategies remains unknown in the context of endocrine resistance in breast cancer.

Here, for the first time we show that in comparison with RAD001, an mTOR kinase inhibitor AZD8055 is significantly superior as a single agent, modulating both mTORC1 and mTORC2 signalling, cell growth and survival in tamoxifen (TamR) and oestrogen deprivation (MCF7-X) resistant cell lines that aim to model clinical relapse following first-line endocrine treatment. Furthermore, we demonstrate that in these endocrine resistant RAD001-resistant models, AZD8055 results in superior growth inhibition when used alongside fulvestrant and is additionally effective alongside anti-hormones during the earlier, endocrine responsive phase of this disease *in vitro*. Cumulatively, these data suggest considerable potential for mTOR kinase inhibitors that target both mTORC1 and 2 to subvert resistance during anti-hormonal management of breast cancer.

## Methods

### Cell culture

The parental ER + breast cancer cell lines were from American Type Culture Collection (ATCC) (Manassas, Virginia, USA) (T47D) or a gift from AstraZeneca (MCF-7) Alderly Park, Macclesfield, (Cheshire, UK). Experimental cells were grown in phenol red-free RPMI-1640 supplemented with 5% FCS (foetal calf serum), penicillin/streptomycin (10 U/ml and 10 μg/ml), fungizone (2.5 μg/ml) and 4 mM glutamine. All cell culture reagents and FCS were from Invitrogen Life Technologies (Fisher Scientific, Loughborough, UK). Cell lines were used within a window of 20 passages. The acquired ER + tamoxifen-resistant cell line, TamR, was derived from MCF-7 cells continuously exposed to 10^-7^ M 4-hydroxytamoxifen (Sigma-Aldrich, Gillingham, Dorset, UK) until emergence (from six months) of a cell line resistant to the growth inhibitory properties of this anti-hormone as previously described [34]. ER + acquired tamoxifen-resistant T47D-tamR cells were also available for this study, similarly derived by our group from T47D cells following continuous exposure to 10^-7^ M 4-hydroxytamoxifen. Stable TamR cells were routinely maintained in phenol-red free RPMI-1640, 5% charcoal-stripped FCS (sFCS) and 10^-7^ M 4-hydroxytamoxifen, with T47D-tamR cells also maintained in the presence of this anti-hormone. The ER + model used for acquired resistance to severe oestrogen deprivation was MCF7-X, derived from MCF-7 cells grown in phenol-red free RPMI containing 5% heat inactivated (65°C, 40 minutes) charcoal stripped FCS (X medium) as described previously [3]. Two ER negative acquired fulvestrant (Faslodex)-resistant cell lines were also available for study generated from MCF-7 (FasR) and T47D (T47D-fasR) cells continuously exposed to fulvestrant (10^-7^ M) for >2 years as previously described [35].

### Growth curves

Cells were seeded overnight at 40,000 cells/well (24-well plate) in their respective growth media. Cells were grown for seven days with 1 nM to 1,000 nM of the mTOR inhibitors AZD8055 or RAD001 (gifts from AstraZeneca) or appropriate vehicle control (dimethyl sulphoxide (DMSO)). Cell growth was evaluated by trypsin dispersion of cell monolayers and cell number was measured using a Coulter Counter. All TamR, MCF-7, MCF7-X, T47D-tamR and T47D-fasR experiments were performed at least in triplicate. In combination studies, fulvestrant was routinely used at 100 nM, a concentration shown previously to be growth inhibitory in TamR and MCF7-X models [2,3]. The growth impact of AZD8055 (0 to 100 nM) alongside oestrogen deprivation (using X cell medium as described above) or 10^-7^ M 4-OH tamoxifen was also evaluated in MCF-7 cells.

### Western blotting

Cell lines were grown to 70% confluence in their respective media and then treated with a concentration range (1 nM to 100 nM) of AZD8055 or RAD001 for 15 minutes to 24 hours. Monolayers were washed with PBS and lysed in ice-cold lysis buffer (50 mM TRIS, 5 mM ethylenediaminetetraacetic acid (EDTA), 150 mM NaCl, 1% Triton-X100 pH 7.5) supplemented with protease and phosphatase inhibitors as previously described [4]. Lysates were clarified by centrifugation (12,000 rpm, 15 minutes, 4°C) and the protein concentration of the supernatant was determined. A total of 20 μg protein was boiled for five minutes in SDS/dithiothreitol (DTT) sample buffer and resolved by SDS-PAGE. Proteins were transferred to nitrocellulose and after blocking for one hour in 5% skimmed milk (10 mM TRIS, 150 mM NaCl, 0.05% Tween 20 pH 7.6), they were incubated overnight with primary antibodies (1:1,000 dilution): mTOR (Cell Signalling Technology, Boston, Massachusetts, USA #2972); P _(ser2481)_ mTOR (Cell Signalling #2974); P_(ser 2448)_mTOR (Cell Signalling #2971); Akt (Cell Signalling #9272); P _(ser 473)_ Akt (Cell Signalling #9271); p70S6k (Cell Signalling #9202); P_(thr389)-_p70S6k (Cell Signalling #9205); S6 ribosomal protein (Cell Signalling #2217); P _(ser235/236)_-S6ribosomal protein (Cell Signalling #2211); 4EBP-1 (Cell Signalling #9644); P _(thr37/46)_4E-BP-1 (Cell Signalling #2855); P _(thr246)_ PRAS40 (Cell Signalling #2997); PRAS40 (Cell Signalling #2610); P-_(thr202/tyr402)_ erk42/44 (Cell Signalling #9101); p44/42 MAPK/erk1/2 (Cell Signalling #9102); ERα (Santa Cruz Biotechnology, Dallas, Texas, USA sc543); P _(ser167)_ER (Upstate Merck Millipore, Billerica, Massachusetts, USA #07-481); and P _(ser118)_ ER (Santa Cruz 12915). Actin (Sigma # A5441, at 1:50,000) was used as a loading control. Blots were washed with TBS/Tween and bound antibodies were detected after one hour incubation with horseradish peroxidase (HRP)-labelled secondary antibodies (1:10,000). Bound proteins were visualised by enhanced chemiluminescence (Pierce, Thermoscientific, Rockford, USA). Where appropriate, signal quantification was performed by densitometry (AlphaEase system) and normalised relative to actin.

### Polymerase Chain Reaction

Total RNA was extracted from monolayers of cells treated for 72 hours with 0 to 100 nM AZD8055 using TRIzol (Sigma) according to the manufacturer’s instructions. Reverse transcription was performed on 1 μg total RNA and PCR was performed as previously described [4]. Oligonucleotide primers were synthesised by Invitrogen and co-amplification was performed with β-actin used as a loading control. Samples treated with oestradiol or fulvestrant were used as a positive control to show ER-regulated modulation of target genes in MCF7-X and TamR cells [3,36]. PCR products were quantified by densitometry (AlphaEase) and normalised relative to actin. The following primers were used in this study:

ß-Actin – GGA GCA ATG ATC TTG ATC T and CCT TCC TGG GCA TGG AGT CCT (202 bp)

Amphiregulin - TCC TCG GGA GCC GAC TAT GAC and GGA CTT TTC CCC ACA CCG (330 bp)

pS2 - CAT GGA GAA CAA GGT GAT CTG and CAG AAG CGT GTC TGA GGT GTC (336 bp)

cyclin D1-GCC TGT GAT GCT GGG CAC TTC ATC TG and TTT GGT TCG GCA GCT TGC TAG GTG AC (358 bp)

c-myc- TTG CAG CTG CTA GAC GCT G and CCA CAT ACA GTC CTG GAT GA (470 bp)

bcl2- CAC CTG TGG TCC ACC TGA C and AGC CAG GAG AAA TCA AAC AGA G (376 bp)

### Immunocytochemistry

Subconfluent monolayers of TamR or MCF7-X cells were treated for one hour (phospho-ERα) or 72 hours (Ki67, ERα, pS2) in their respective growth media in the presence of AZD8055 (1 to 100 nM) on 3-aminopropyltriethoxysilane coated glass coverslips. Staining for the proliferation marker Ki67 (MIB1 antibody) was performed on cells fixed in 3.7% formaldehyde/0.15 M NaCl for ten minutes, five minutes in 100% ethanol with a final wash in PBS before assay. Staining for phosphorylated ERα (ser167 or ser118 sites), pS2 and total ERα was performed on cells optimally-fixed for fifteen minutes with 3.7% formaldehyde in PBS, five minutes PBS, five minutes methanol (-20°C), five minutes acetone (-20°C) and five minutes PBS or with 2% paraformaldehyde with 20 mM orthovanadate for twenty minutes followed by 2 x five minutes PBS washes (ERα 118). Coverslips were blocked with PBS/0.02%Tween for five minutes and incubated with 1:150 MIB1 (M7074 Dako Ltd, Ely, Cambridgeshire, UK) in PBS for one hour, or with 1:175 ERα (AB ER clone 6 F11 Vector Laboratories, Peterborough, UK) for 90 minutes; 1:100 ERα 167 (Upstate #07-481); 1:400 pS2 (Novocastra, Leica Biosystems, Newcastle-upon-Tyne, UK) for 90 minutes or 1:25 ERα 118 (Cell Signalling # 2515) overnight at room temperature. Unbound antibody was removed by washing with PBS. The EnVision (Dako) system was used for visualization (one to two hours at room temperature). Coverslips were washed in PBS and detected with diaminobenzidine tetrahydrochloride (DAB) and hydrogen peroxide chromogen substrate (Dako) and counterstained with methyl green. Immunostaining was evaluated at 20× magnification using an Olympus BH-2 light microscope and representative photographs were taken. For Ki67, estimates of percentage of cells deemed positive versus negative/equivocally-stained were determined from five fields of view for each coverslip (20x magnification) from three independent experiments.

### Sytox green viability assay

A Sytox green viability assay was modified from Jones and Singer [37]. Cells were seeded into 96-cell plates (5,000 cells/well) and left to adhere overnight (37°C, 5% CO_2_). After 24 hours (day 0), Sytox green, a cell impermeant green-fluorescent nucleic acid stain (Invitrogen), was added to each control well (n = 8) (final concentration 1:25,000) and after one hour the number of cells with a compromised plasma membrane (that is, late apoptotic and necrotic cells) that had taken up the sytox green was counted (number/well) using a fluorescent microscope. Cell membranes were permeabilised overnight with 0.25% saponin (Sigma) in the presence of sytox green, and then total cell number/well was counted. The remaining experimental cells were cultured for 72 hours in the presence of AZD8055 (0 to 100 nM), then the sytox green assay was performed to give a count for dead and total cell numbers as described above. Data were subsequently analysed by comparing live cell counts on day 0 with live cell counts on day 3. Cell death was considered to have occurred when the viable cell number fell below the day 0 pre-treatment control number. Each treatment was assessed from eight replicates and three independent experiments.

### Migration assay

Pore inserts (8 μm, Costar #3422) were incubated with 300 μl sterile fibronectin (Sigma) in PBS (10 μg/ml) for two hours at 37°C. Excess fibronectin was removed from the bottom of the insert by washing in PBS. Inserts were air dried for 15 minutes, then 650 μl cell culture medium +/- 25 nM AZD8055 was placed in the well and the insert suspended in it. A total of 40,000 TamR cells in normal growth medium were added to the insert and the plate was incubated for 24 hours at 37°C. Cells on the insert were fixed with 3.7% formaldehyde (15 minutes). Cells on the inside of the insert were removed with a cotton swab and cells that had migrated to the lower side of the insert membrane were stained with 0.5% crystal violet for 30 minutes. Excess stain was removed by repeated washing with distilled water. Migrated cells were counted under an inverted light microscope over five fields of view (x20). Each experiment included triplicate wells and the experiment was repeated twice.

### Statistics

Statistical analyses were carried out using a two sided t-test and ANOVA with *post hoc* test. *P* <0.05 was considered significant.

## Results

### Differential effects of RAD001 and AZD8055 on proliferation and signalling in acquired endocrine- resistant models

**Figure 1:**
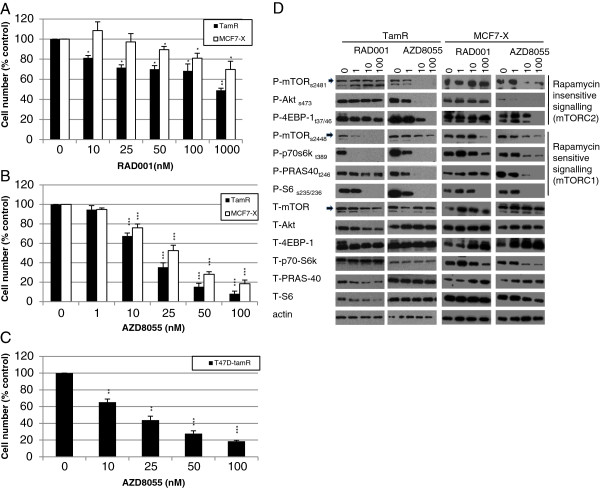
**Comparison of RAD001 and AZD8055 effect on cell growth and mTORC1/2 signalling pathways in TamR and MCF7-X cells.** TamR (black squares) and MCF7-X cells (white squares) were grown for seven days in the presence of RAD001 (0 to 1,000 nM) **(A)** or AZD8055 **(B)** (0 to 100 nM) and cell number determined by Coulter Counting. The effect of AZD8055 on seven days growth of tamoxifen-resistant T47D-tamR cells similarly determined is shown in **(C)**. Results expressed as% control are the mean (+/- SEM) of three to five independent experiments. *P <0.05 versus appropriate cell line control (0), **P <0.01 versus appropriate cell line control (0), ***P <0.001 versus appropriate cell line control (0). Western blot of 70% confluent TamR and MCF7-X cells treated for one hour with RAD001 or AZD8055 (0 to 100 nM) **(D)**. Blots were probed with phospho- and total antibodies for mTORC1 (rapamycin sensitive) and mTORC2 (rapamycin insensitive) signalling pathways. Blot shown is representative of at least two independent experiments.

The allosteric mTOR inhibitor RAD001 (everolimus) was a relatively poor inhibitor of growth measured over seven days in MCF7-derived tamoxifen-resistant cells (TamR) with an IC_50_ of 950 nM. In long-term oestrogen deprived (MCF7-X) resistant MCF-7 cells, RAD001 was found to be even less potent (*P* <0.05) with an IC_50_ >1 μM (Figure 1A). In contrast, the mTOR kinase inhibitor AZD8055 at 10 to 100 nM was a very effective inhibitor of growth in TamR cells (*P* <0.001) with an IC_50_ of 18 nM. AZD8055 10 to 100 nM also substantially (*P* <0.001) inhibited growth of the MCF7-X cell model, with an IC_50_ of 24 nM (Figure 1B), although MCF7-X cells were significantly less sensitive than the TamR cells to AZD8055 when examined at 25 nM (*P* = 0.035) and 50 nM (*P* =0.019). Additional studies performed in a tamoxifen-resistant T47D-derived model (T47D-tamR) further confirmed that AZD8055 was highly growth inhibitory (IC_50_ 19 nM) in acquired endocrine-resistant cells (Figure 1C).

Despite having a poorer effect on cell growth, one hour treatment with RAD001 was still shown to inhibit mTORC1 (rapamycin sensitive) associated signalling pathways with TamR cells being slightly more sensitive to RAD001 than MCF7-X cells (Figure 1D). In both cell lines, RAD001 at 100 nM caused a reduction in mTOR phosphorylation at s2448, which has previously been described as the site predominantly associated with the mTORC1 complex [38]. Downstream p70S6K phosphorylation in TamR was undetectable after treatment with 1 nM RAD001 and pS6 activity was inhibited with RAD001 >10 nM. These mTORC1 downstream pathways were less sensitive to RAD001 in MCF7-X cells, but phosphorylation of p70S6K and pS6 were still inhibited by 100 nM RAD001. However, in both models, there was no impact of one hour treatment with RAD001 on pPRAS40. RAD001 was a poor inhibitor of mTORC2 (predominantly rapamycin-insensitive) pathways in both TamR and MCF7-X cells, indicated by its failure to significantly reduce both s473 Akt phosphorylation and mTOR phosphorylation at s2481, a site known to be associated with mTORC2 [38]. In both TamR and MCF7-X cells, RAD001 also failed to inhibit 4EBP-1 phosphorylation on the t37/46 site which has previously been described as rapamycin-insensitive [12].

In contrast to RAD001, one hour treatment with AZD8055 inhibited pathways associated with both mTORC1 and mTORC2 signalling in both TamR and MCF7-X endocrine-resistant cell lines (Figure 1D). At concentrations from 1 to 100 nM, the inhibition of mTORC1 pathway elements, p-p70s6kinase and p-S6 ribosomal protein was similar or superior with AZD8055 to that seen with RAD0001. While inhibition of p-PRAS40 was not detected after one hour treatment with RAD001, PRAS40 phosphorylation was eliminated by 100 nM AZD8055 in both TamR and MCF7-X cells. The biggest difference was seen with the mTORC2 associated signalling caused by AZD8055 with reduced activation of s2481 p-mTOR, complete inhibition of p-Akt by AZD8055 at 1 to 10 nM and at concentrations >10 nM complete inhibition of 4EBP-1 at the rapamycin insensitive site t37/46. There was no consistent effect across replicate preparations on total protein expression over one hour treatment with either RAD001 or AZD8055.

### AZD8055 effect on mTORC1 and mTORC2 signalling in TamR and MCF7-X cells is rapid and sustained

Since superior growth blockade and mTORC1/mTORC2 signalling inhibition was induced by AZD8055 in the endocrine resistant cancer cells, our subsequent detailed studies focused entirely on AZD8055. We investigated the sustainability of the AZD8055 signalling response and the inhibitory impact of AZD8055 on cell proliferation and survival in the TamR and MCF7-X resistant models.

**Figure 2:**
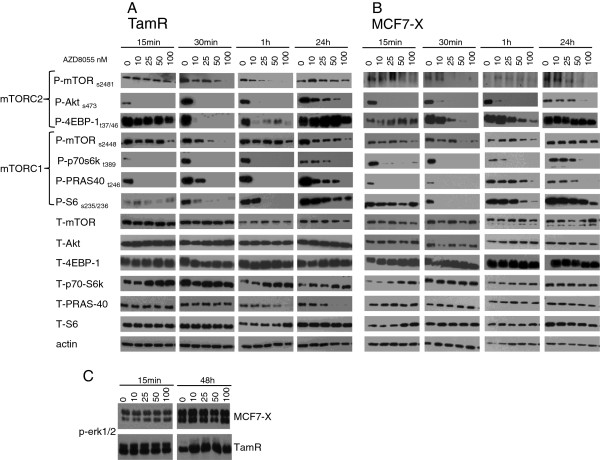
**Time course of AZD8055 effect on mTORC1 and TORC2 signalling pathways in TamR and MCF7-X cells.** Western blots of 70% confluent TamR **(A)** and MCF7-X **(B)** cells treated for 15 minutes to 24 hours with AZD8055 (0 to 100 nM). Blots were probed with phospho- and total antibodies for mTORC1 and two signalling pathways. In both cell lines mTORC1 and mTORC2 signalling pathways were rapidly inhibited by AZD8055. Blots shown are representative of at least two independent experiments. Western blots of 70% confluent TamR and MCF7-X cells treated for 15 minutes to 48 hours with AZD8055 (0 to 100 nM) probed with antibodies for MAPK activity (p-erk1/2) showing no effect **(C)**. Blots shown are from representative experiments.

Initial studies showed that within one hour AZD8055 (10 to 100 nM) inhibited both mTORC1 and mTORC2 signalling pathways similarly in both TamR and MCF7-X cells. Further studies were performed over a time course from 15 minutes through to 24 hours**.** Western blotting showed that mTORC1 and mTORC2 signalling in TamR and MCF7-X cells were both extremely sensitive to AZD8055 with 30 minutes treatment with 50 nM AZD8055 demonstrating strong inhibition of mTOR at sites s2448 and s2481 in both resistant cells (Figure 2A-B). AZD8055 at a concentration of 10 nM completely inhibited phosphorylation of Akt (s473), p70s6K (t389) and PRAS40 (t246) within 15 minutes. Reduction of phosphorylation of pS6 (s235/236) and 4EBP-1 (t37/46) was slightly slower, with complete inhibition being detected in TamR cells after 30 minutes of AZD8055 treatment, although p4EBP-1 was slightly less sensitive to inhibition in MCF7-X cells. The inhibitory effects of AZD8055 at 100 nM were generally sustained throughout the time course, with the exception of p4EBP-1 and p-mTOR (s2448) in MCF7-X cells, although by 24 hours some recovery of signalling pathways (where expression returned to basal levels) was seen at the lower concentrations of AZD8055 examined. As expected, following 15 minutes to 48 hours treatment, we also observed that AZD8055 (10 to 100 nM) did not modulate the activation of p-erk1/2 in both TamR and MCF7-X cells (Figure 2C).

### Effect of AZD8055 on TamR and MCF7-X proliferation, cell survival and migratory behaviour

**Figure 3:**
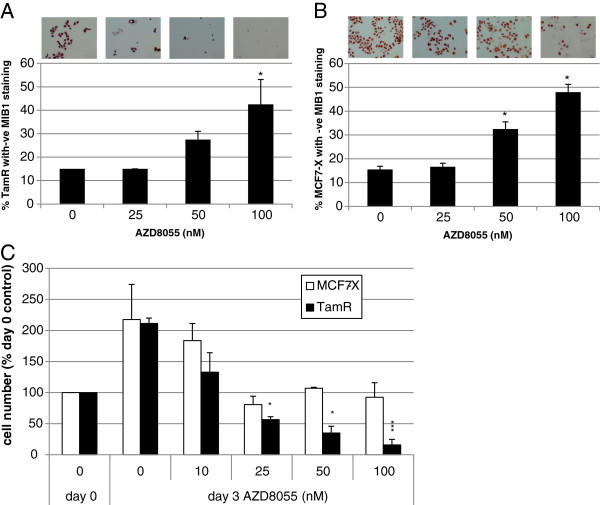
**Effect of AZD8055 on proliferation and viability in TamR and MCF7-X cells.** Immunocytochemical evaluation of MIB1 proliferation marker (Ki67) in TamR **(A)** and MCF7-X cells **(B)** treated for three days with AZD8055 (0 to 100 nM). Multiple fields of view (x20) were assessed for% cells expressing no/equivocal MIB1 staining. Results are from three independent experiments. * *P* <0.05 versus untreated control (ANOVA with *post-hoc* test). Sytox green impermeable nuclear stain was used to measure live cell count in TamR and MCF7-X cells in a viability cell assay before and after three days treatment with AZD8066 **(C)**. After three days in the presence of 25 nM AZD8055, TamR live cell number fell below the pre-treatment count indicating some cell death with this agent. Live cell count (total minus dead cells) was a mean of eight replicates in three independent experiments. *P <0.05, ***P <0.001 for three days treatment with AZD8055 versus appropriate day 0 control ANOVA with *post-hoc* test. ANOVA, analysis of variance.

**Figure 4:**
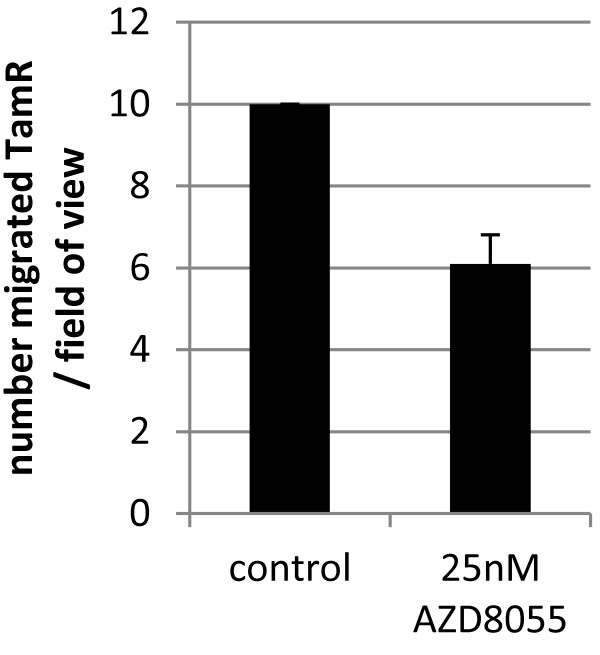
**Effect of AZD8055 on migration in TamR cells.** Twenty-four hours migration was measured over an 8 μm pore membrane coated with fibronectin in the presence or absence of 25 nM AZD8055. Results shown are from a representative experiment (n = 2).

The impact of AZD8055 on TamR and MCF-7-X cell proliferation was monitored using MIB1 Ki67 staining. Three days treatment with 50 nM AZD8055 reduced Ki67 staining in both TamR and MCF7-X cells and after treatment with 100 nM 40% to 50% of all cells were deemed negative for MIB1 indicating a significant (*P* <0.05) exit from the cell cycle (Figure 3A-B). These MIB1 results indicated that the dual mTORC1/2 inhibitor AZD8055 was acting to partially inhibit cell proliferation in TamR and MCF7-X cells. It was investigated whether cell death also contributed to reduced cell numbers in the presence of a concentration range of AZD8055. The sytox green viability assay showed that in TamR cells AZD8055 was not only cytostatic, but at concentrations >25 nM, AZD8055 induced significant cell death with the TamR viable cell number falling to approximately 50% below the initial seeding number (*P* <0.05). In contrast, in MCF7-X cells there was evidence from sytox green assays that while an anti-proliferative effect occurred this was without any significant cell death with AZD8055 when used as a single agent (Figure 3C). TamR cells (but not MCF7-X) have increased migratory ability compared to parental MCF-7 cells [39]. Although numbers of migrated TAMR cells were very modest, following 24 hours treatment with AZD8055 TamR migration was shown to be reduced by 40% showing that dual mTORC1/2 blockade has the capacity to impact on both resistant tumour cell growth and aggressiveness (Figure 4).

### Investigation of any cross talk between ER and mTOR signalling targeted by AZD8055 in TamR and MCF7-X cells

**Figure 5:**
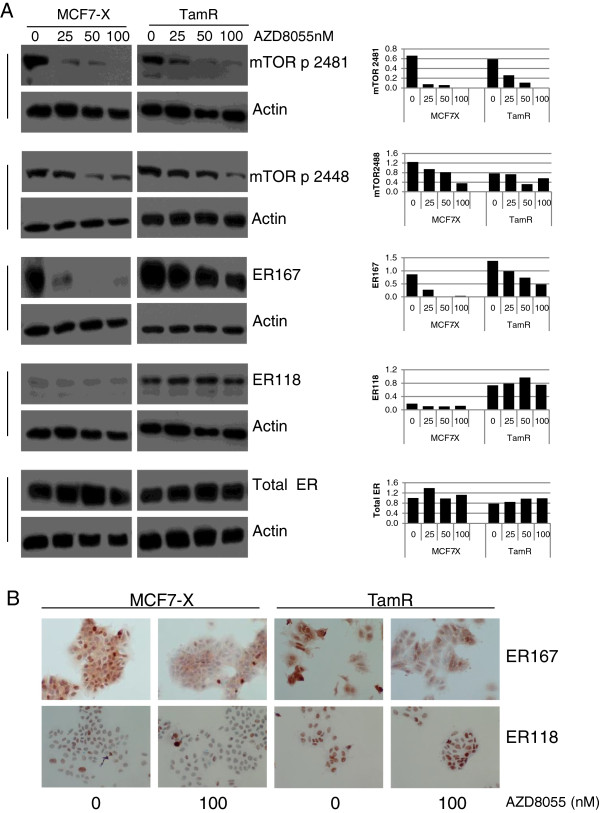
**One hour treatment with AZD8055 affects pER**_**ser167 **_**protein expression in TamR and MCF7-X cells.** Western blot of 70% confluent TamR and MCF7-X cells treated for one hour with AZD8055 (25 to 100 nM). Images show blots probed for pER (s118 and s167), total ER, mTOR phosphorylation on s2481 and s2448 to demonstrate mTOR inhibition and actin to indicate equal loading. The accompanying bar charts show densitometric analysis of western blot data normalised to actin. pER_ser167_ but not pER_ser118_ protein was inhibited in MCF7-X and TamR cells. Blots are representative of two independent experiments **(A)**. Accompanying immunocytochemical images confirm the effect of AZD8055 on pER_ser167_ but not pER_ser118_ protein expression in TamR and MCF7-X cells. Immunocytochemistry was performed on TamR and MCF7-X cells fixed after treatment +/- AZD8055 (100 nM) for one hour and cells were stained with antibodies for pER_ser118_ and pER_ser167_**(B)**. At least five fields of view were examined for staining and representative images (x20) shown. Results are shown from at least three independent experiments.

Both TamR and MCF7-X cells were derived from oestrogen-dependent MCF-7 breast cancer cells that have acquired tamoxifen or oestrogen deprivation resistance, respectively, but still grow in an ERα dependent manner [40]. In the TamR cell line, it is already known that there is prominent cross-talk between erk1/2 and phosphorylation of the ER s118 site in the ER AF-1 domain [36]. In MCF7-X cells, MAPK and PI3K/Akt also have the capacity to cross-talk with ER at pER_ser118_ and pER_ser167_, respectively [3]. Activation at such ER sites by cross-talk can contribute to driving transcription of oestrogen/ER regulated genes, notably amphiregulin which plays a part in the growth of TamR cells [36,41] and also pS2 expression in MCF-7X cells [3]. The possible contribution of cross-talk between mTOR signalling and ER in these models of tamoxifen or oestrogen deprivation resistance was thus investigated using AZD8055 in the current study. Western blotting of MCF7-X and TamR confirmed prominent basal ER phosphorylation levels at ser118 and 167 in the latter model. Both models treated for one hour with AZD8055 (0 to 100 nM) showed, in conjunction with downregulation of mTOR activity at s2448 and s2481, a concentration dependent inhibition of ER phosphorylation at s167 (Figure 5A). Total ER and phosphorylation of ER at s118 were not significantly affected by AZD8055. Parallel immunocytochemistry (ICC) confirmed that nuclear pER_ser167_, but not pER_ser118_, in both MCF7-X and TamR cells was reduced after one hour exposure to 100 nM AZD8055 (Figure 5B).

**Figure 6:**
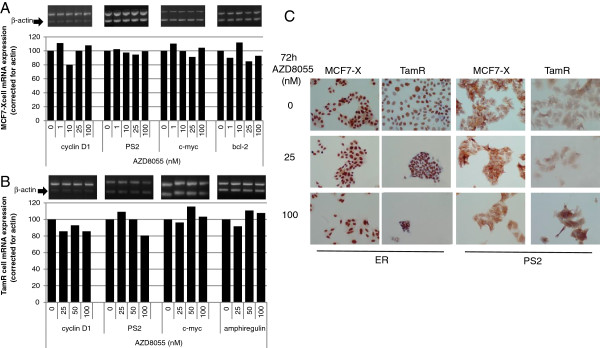
**AZD8055 does not significantly affect ER regulated gene expression in MCF7-X and TamR cells.** RNA was extracted from MCF7-X **(A)** and TamR **(B)** after 72 hours treatment with AZD8055 (0 to 100 nM) and gene expression analysed by semi-quantitative PCR. The image shows an ethidium bromide stained 2% agarose gel with the upper band showing cyclin D1, pS2 c-myc, bcl2 (only expressed in MCF7-X) and amphiregulin (only expressed in TamR) and the β-actin PCR product (lower band). The bar charts show actin-normalised values for the ER regulated genes which are not significantly affected by AZD8055. Data are representative of two independent experiments. Accompanying immunocytochemical images confirm that 72 hours treatment with a concentration range of AZD8055 (0 to 100 nM) does not significantly affect pS2 or total ER protein expression in MCF7-X or TamR cells **(C)**. Images shown (x20) are representative of three independent experiments.

**Figure 7:**
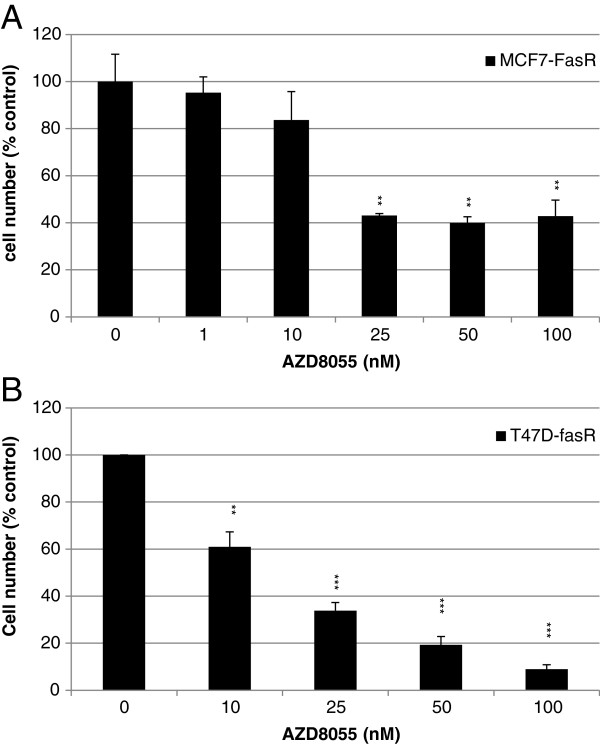
**AZD8055 effect on growth of ER- acquired fulvestrant-resistant MCF7 (FasR) and T47D cells (T47D-fasR).** FasR **(A)** and T47D-fasR **(B)** cells were grown for seven days in the presence of AZD8055 (0 to 100 nM) and cell number determined by Coulter counting. Results expressed as% control. **P <0.01, ***P <0. 001.

Potential interplay between mTOR blockade using AZD8055 and ER signalling was further investigated by PCR examination of the ER regulated gene pS2 as well as several ER-regulated genes more closely related to breast cancer cell growth: amphiregulin (important in TamR cells; [36], bcl2 (expressed in MCF7-X only), c-myc and cyclinD1. mRNA expression of these ER regulated genes was measured after 72 hours treatment with a concentration range of AZD8055 (1 to 100 nM) but failed to show any significant altered gene transcription in TamR or MCF7-X cells (Figure 6A-B). ICC in TamR and MCF7-X cells confirmed that 72 hours treatment with 1 to 100 nM AZD8055 caused a concentration dependent reduction in cell number but did not alter expression of pS2 or ER protein in TamR or MCF7-X cells (Figure 6C). While only examining a limited panel of ER-regulated genes, these data do suggest that the mTOR inhibitor impact was independent of changes in ER-regulated transcriptional events and, hence, that mTOR and ER signalling are unlikely to be closely interactive in these acquired resistant models. The capacity for ER independent impact of AZD8055 was further supported by the demonstration that 25 nM AZD8055 also inhibited growth by 60% (IC_50_ 20 nM) in an ER negative acquired fulvestrant-resistant cell line derived from MCF7 cells (FasR) (Figure 7A). This observation was also supported by AZD8055 growth studies in a T47D-derived ER- acquired fulvestrant-resistant line (T47D-fasR) with an IC_50_ of 18 nM (Figure 7B).

### AZD8055 and fulvestrant in combination enhance growth inhibition in TamR and MCF7-X cells

**Figure 8:**
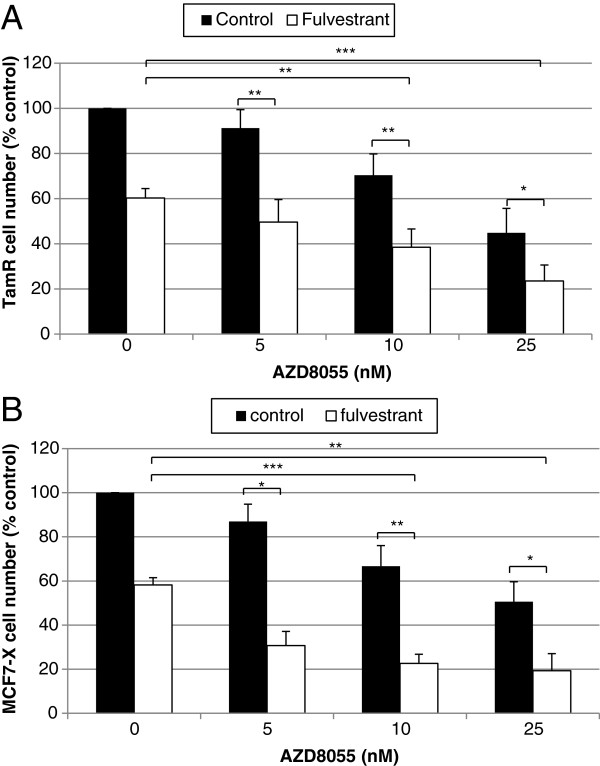
**Greater inhibition of TamR (A) and MCF7-X (B) cell growth by the pure anti-oestrogen fulvestrant is apparent when the drug is used in combination with AZD8055.** TamR cells were grown for seven days in the absence of tamoxifen. TamR and MCF7-X cells were then grown for seven days in the presence of fulvestrant (10^-7^ M) and AZD8055 (0 to 25 nM). Cells were counted and the results expressed as a percentage of the untreated control. Results are means of four independent experiments. Statistical analyses used ANOVA with a *post hoc* test to compare doses of AZD8055 plus fulvestrant versus AZD8055 alone and AZD8055 plus fulvestrant versus the fulvestrant alone control (0). *P <0.05, **P <0.01, ***P <0. 001. ANOVA, analysis of variance.

When ER positive breast tumours acquire resistance to an anti-hormone, an alternative anti-endocrine therapy can often be used successfully second-line, although resistance invariably emerges. In keeping with this, we have previously shown that the pure anti-oestrogen fulvestrant is growth-inhibitory in TamR [2] or MCF7-X cells *in vitro*[3], but growth inhibition is only partial, with the cells subsequently acquiring resistance to fulvestrant. We have investigated whether co-treating with a further anti-hormonal measure (fulvestrant) alongside an mTOR kinase inhibitor could offer an improved second-line treatment for endocrine-resistant cells versus either strategy alone. This was also important to evaluate since AZD8055 appears to be inhibitory independently of any substantial impact on ER-regulated events in our acquired endocrine resistant models. When TamR cells were treated for seven days with 25 nM AZD8055 in combination with fulvestrant (100 nM) there was a further 60% decrease in growth above that caused by fulvestrant (*P* <0.001) and a 50% enhancement of growth inhibition compared to AZD8055 alone (*P* <0.05) (Figure 8A). A similar improved anti-tumour effect was also observed in MCF-7X cells co-treated with AZD8055 and fulvestrant (Figure 8B) in which 25 nM AZD8055 caused a further 60% decrease in growth above fulvestrant (*P* <0.01) or AZD8055 alone (*P* <0.05). These results suggest that an mTOR kinase inhibitor (exemplified here by AZD8055) plus anti-oestrogen fulvestrant combination could have potential as a superior second-line treatment for endocrine resistant breast cancers that do not respond well to the rapalogue everolimus.

**Figure 9:**
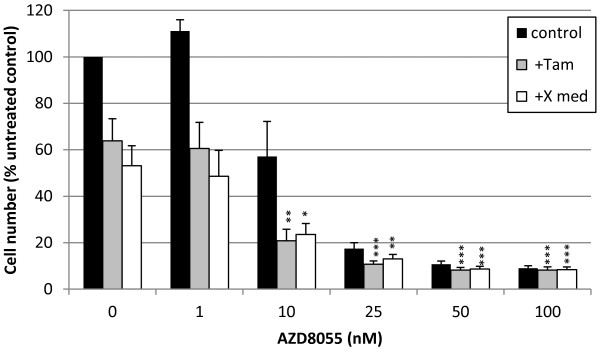
**Greater inhibition of MCF-7 cell growth is apparent when AZD8055 is used in combination with tamoxifen or oestrogen deprivation.** Seven days growth of oestrogen dependent MCF-7 cells was compared in standard control medium (black squares), in the presence of 10^-7^ M tamoxifen (grey square) or oestrogen-deprived medium (X cell medium) (white squares) with a concentration range of AZD8055 (0 to 100 nM). Triplicate wells of cells were counted by Coulter Counter and the results expressed as a percentage of the untreated control cells. Combination with 10 nM AZD8055 significantly increased growth inhibition in the presence of tamoxifen or oestrogen-deprived medium by 66% and 56%, respectively, versus anti-hormone alone. *P <0.05, **P <0.01, ***P <0.001 versus appropriate anti-hormone control in the absence of AZD8055 (0). Results shown are means of three independent experiments. Statistical analysis was performed with ANOVA and *post hoc* test. ANOVA, analysis of variance.

Finally, while seven day treatment with AZD8055 was also a good inhibitor of growth in anti-oestrogen sensitive MCF-7 parental cells with an IC_50_ of 12 nM (Figure 9), a superior growth inhibition could again be obtained by co-treatment with AZD8055 and either 4-OH-tamoxifen (10^-7^ M) or severe oestrogen deprivation (X medium containing charcoal stripped and heat treated FCS). The anti-tumour effect was increased by 66% (*P* <0.01) and 56% (*P* <0.05), respectively, by combination with 10 nM AZD8055 versus the anti-hormone treatment alone (Figure 9). These findings suggest that in combination with anti-hormone therapy, mTOR kinase blockade could also provide a first-line treatment strategy to inhibit endocrine responsive disease more effectively and thereby hinder acquisition of resistance in breast cancer.

## Discussion

### Endocrine resistant breast cancer and clinical status of mTOR targeting with rapalogue RAD001

Acquisition of resistance to endocrine therapy remains a major problem in the treatment of ER + breast cancer and using inhibitors to target de-regulated growth factor signalling pathways, such as mTOR, has become an important therapeutic approach currently under intense clinical evaluation. Initial preclinical studies demonstrated that tamoxifen resistance is mediated in part by mTOR signalling [42]. This contributed towards the rationale for successful clinical studies where rapalogue treatment was combined with aromatase inhibitor (BOLERO-2) or tamoxifen (TAMRAD) therapy [26,27,43,44] which resulted in approval of the rapalogue everolimus (Afinitor, RAD001), in combination with the steroidal aromatase inhibitor exemestane (Aromasin), for treatment of post-menopausal women with advanced ER+/HER2- breast cancer progressing on a non-steroidal aromatase inhibitor. Critically, however, although the BOLERO-2 trial showed that the objective response rate was improved for everolimus/antihormone combination versus antihormone alone, no patients showed complete response and some patients remained refractory to this rapalogue therapy or developed resistance during treatment [26]. The signalling pathways that limit the impact of rapalogues in endocrine resistant breast cancer have to date been largely undefined. Here, we have studied the use of the mTOR kinase inhibitor AZD8055 as a potential treatment for acquired endocrine-resistant breast cancers including those refractory to rapalogue treatment.

### Importance of mTORC2/AKT signalling in acquired endocrine resistant models resistant to rapalogue RAD001

Our study has predominantly focussed on MCF7-X and TamR cells as *in vitro* models that aim to represent clinical relapse after first line oestrogen deprivation or tamoxifen treatment, respectively [2,3]. Interestingly, we found that MCF7-X cell growth was completely, and TamR cells partially, resistant to inhibition by RAD001 (everolimus, IC_50_ ≥1uM), despite inhibition of target TORC1 signalling (mTORC_ser2448_, p70S6k and pS6). Similar growth and signalling effects have been reported by others in MCF-7 cells with acquired tamoxifen-resistance [45]. Critically, in our study RAD001 failed to inhibit the mTORC2 signalling complex which is a major regulator of Akt activity [46]. Thus, p-mTOR_ser2481_ (mTORC2), p-Akt_ser473_ and also growth, which is in part AKT-driven in TamR and MCF7-X cells [3,4], were not inhibited by this agent. Reports of rapamycin-sensitivity in long-term oestrogen deprived (LTED) MCF-7 cells from other groups [45,47-50] have ranged from being modest [48] to very sensitive [49], with increased HER2 expression invariably reported to be a predictor for their increased RAD001 sensitivity [45,49]. Oestrogen deprivation-resistant MCF7-X cells differ in that they do not have up-regulation of HER2 (or IGF1R) [3], and indeed such growth factor receptors only rarely increase in clinical endocrine relapse samples [51]. It is thus probable that some dependence on the PI3K/Akt pathway in MCF7-X cells coupled with only low HER2 activity cumulatively results in their prominent growth-insensitivity to RAD001.

### Rapalogue insensitive acquired endocrine resistant cells retain sensitivity to mTOR kinase inhibition

Given their substantial RAD001 growth insensitivity, TamR and MCF7-X cells could comprise useful models to understand everolimus resistance in tamoxifen or oestrogen deprivation-resistant patients and to determine improved treatments. In this regard, our findings suggest that inhibition of both TORC2/Akt and TORC1 may be critical. We have shown that growth of cancer cells unresponsive to an allosteric mTOR inhibitor can still be sensitive to mTOR kinase blockade, with AZD8055 substantially inhibiting growth in TamR and MCF7-X cells (IC_50_ 18 and 24 nM, respectively). To the best of our knowledge, this is the first report suggesting the potential for mTOR kinase inhibitors in rapalogue-(RAD001) insensitive ER+/HER2- breast cancer cells with acquired endocrine resistance. Clearly, it should be remembered that there are limitations to studies based on homogeneous cell cultures because such modelling cannot reflect the breadth of clinical heterogeneity of ER + disease or microenvironment impact [52,53]. However, experimental work has shown that resistant cell lines can reflect features seen in some clinical breast cancer samples [54,55] and our additional results here are encouraging in that they show AZD8055 is also highly growth-inhibitory in a further ER + acquired endocrine-resistant model, T47D-tamR (IC_50_ 19 nM).

Our TamR and MCF7-X signalling studies indicate that the difference in the mechanism of action of these drugs enables AZD8055 to target mTORC2 signalling in addition to mTORC1; thus, basal p-Akt_ser 473_ is rapidly and substantially inhibited by AZD8055 in both models but is unaffected by RAD001. Furthermore, inhibition of a negative feedback loop downstream of mTORC1 [22,24] can result in induction of Akt activity in patients treated with everolimus [18]. This event may limit the effectiveness of mTORC1-targeting therapy since it can be associated with shortened time to progression in patients [56,57]. However, in both TamR and MCF7-X cells, basal Akt _ser473_ inhibition by AZD8055 was sustained with no up-regulation over 24 hours treatment. A similar response to AZD8055 has been reported in further breast cancer cell lines [12], while clinically a close analogue of AZD8055, AZD2014, also inhibits pAKT, pS6 and p4EBP-1 in some tumours [58].

AZD8055 has previously been reported to induce cell death and autophagy in lung and leukaemia cancer cells [59-61], contrasting rapalogues that are often poor inducers of cell death [12,59]. In the present study, some cell death was induced in TamR but not in MCF7-X cells by AZD8055. Since bcl-2 mRNA was detected basally in MCF7-X, but not in TamR cells, this anti-apoptotic factor may contribute towards the somewhat-reduced AZD8055-sensitivity in MCF7-X versus TamR cells. Interestingly, rapamycin-resistant tumours have previously been reported to express high levels of bcl-2 [62,63]. The increased effectiveness of AZD8055 over RAD001 in TamR and MCF7-X cells may also relate to the reported ability of mTOR kinase inhibitors to deplete overall protein synthesis more strongly than rapamycin [64], where inhibition of 4E-BP1 phosphorylation at rapamycin resistant sites t37 and 46 [12] with AZD8055 may increase inhibition of cap-dependent translation [61,65]. Interestingly, AZD8055 showed a superior impact on 4E-BP1 phosphorylation in TamR versus MCF7-X cells, which may contribute towards increased AZD8055 sensitivity in the former model.

### Sensitivity to mTOR kinase inhibitor can occur independently of ER in acquired endocrine resistant cells

Clinically, acquired endocrine-resistant tumours often respond to second-line antihormonal therapy and TamR and MCF7-X cells similarly retain ER dependency, responding to fulvestrant challenge [2,3]. In endocrine-resistant cancers, nuclear ER activity can be driven in a ligand-independent manner via cross-talk with growth factor protein kinase cascades including MAPK and PI3K/Akt with emerging evidence for a role of mTOR [43]. Thus, rapalogue treatment has been reported to inhibit pER_ser167_ in LTED MCF-7 cells and in a tamoxifen and fulvestrant-resistant MCF-7-derived line R-MVLN [49,66], while S6kinase (S6K), a downstream TORC1 target, is also able to phosphorylate a consensus motif at pER_ser167_[67,68]. Although our results have also provided some support of cross-talk via ER phosphorylation in MCF7-X and TamR cells, where pER_ser167_ was rapidly inhibited by the mTOR kinase inhibitor AZD8055, extensive PCR investigation failed to demonstrate any significant inhibitory effect of AZD8055 on ER-regulated gene transcription. We have also been unable to show a convincing impact of AZD8055 on basal or oestradiol-stimulated oestrogen response element activity in MCF7-X and TamR using reporter gene construct studies (data not shown). The contribution of ER phosphorylation at individual sites is generally underexplored, although phosphorylation of either ser^167^ or ser^118^ in the AF1 domain of ERα can exert only a small effect on gene transcription [69]; hence, the significance of ER_ser167_ inhibition with AZD8055 in TamR and MCF7-X cells remains unclear. Significantly, in TamR cells, EGFR/MAPK signalling is a predominant driver of pER_ser118_[36] and MAPK also has some capacity in maintaining pER_ser118_ activity in MCF7-X cells [3]. The observation that activity of both pER_ser118_ and MAPK were refractory to AZD8055 may thus explain the apparent inability of the drug to impact on genomic ER function in these endocrine-resistant cells. Clearly, AZD8055 appears able to promote its growth inhibitory effects in an ER independent manner in TamR and MCF7-X cells, an outcome supported by the observation that it retains substantial growth-inhibitory activity in two acquired fulvestrant-resistant cell lines that have lost all ER expression (MCF-7-derived FasR and also T47D-fasR) [35]. Interestingly, rapalogues have also been shown to have favourable activity in triple-negative breast cancer cells [70].

### Superior impact of mTOR kinase inhibitor alongside further ER blockade (fulvestrant) in acquired endocrine resistant models

We have previously shown that although ER remains an important growth contributor in TamR and MCF7-X cells, fulvestrant responses are incomplete in these models and resistance subsequently emerges during treatment [2,3,36]. This finding mirrors relapse during second line endocrine treatment that occurs in many patients. Reports in various cell lines have shown that co-treatment with everolimus and endocrine therapy can exert additive or synergistic growth inhibitory effects [42,43,71,72]. Importantly, AZD8055 significantly improved the anti-tumour effect of fulvestrant in both TamR and MCF7-X cells, suggesting that such combination treatment might prove valuable in breast cancer after initial endocrine failure. Moreover, since our TamR and MCF7-X models were also RAD001 refractory, it is feasible that the value of such combination therapy might extend to patients who are refractory to combined treatment with everolimus plus exemestane or tamoxifen [26,27]. Our pre-clinical findings are promising given that trials are ongoing in advanced breast cancer patients using fulvestrant in combination with the mTOR kinase inhibitor AZD2014 (*ClinicalTrials.gov*: NCT01597388). Finally, it is noteworthy that in the parental MCF-7 line, we also observed a greater anti-tumour effect with AZD8055 alongside tamoxifen or oestrogen-deprivation. As such, co-treatment may additionally have some capacity to hinder development of resistance. The efficacy of everolimus alongside endocrine agents in the adjuvant setting is currently being explored in ER+/HER2- patients at high risk of relapse (*ClinicalTrials.gov:* UNIRAD), and based on our pre-clinical findings here, evaluation of early combination treatment using an mTOR kinase inhibitor may be equally worthy of exploration where it may help to delay or prevent acquisition of endocrine resistance.

## Conclusions

Our findings using endocrine-resistant breast cancer cell lines demonstrate for the first time that dual targeting of mTORC1 and mTORC2/AKT signalling with an mTOR kinase inhibitor (AZD8055) can be effective even under conditions in which the allosteric mTORC1 inhibitor RAD001 (everolimus) fails to control growth. Moreover, combined treatment with AZD8055 alongside anti-hormones provides a particularly potent growth inhibitory strategy both for the TamR and MCF7-X models and for endocrine responsive MCF-7 cells.

## Abbreviations

4E-BP1: eukaryotic translational initiation factor 4E binding protein 1; ANOVA: analysis of variance; BC: breast cancer; bp: base pair; DMSO: dimethyl sulphoxide; EGFR: epidermal growth factor receptor; ER: oestrogen receptor; FCS: foetal calf serum; HER2/erbB2: human epidermal growth factor receptor-2; ICC: immunocytochemistry; IGF-1R: insulin-like growth factor receptor 1; LTED: long-term oestrogen deprived; MAPK: mitogen activated protein kinase.; mTOR: mammalian target of rapamycin; p70S6K: p70 ribosomal S6 kinase, S6K S6 kinase; PBS: phosphate-buffered saline; PCR: polymerase chain reaction; PI3K: phosphtidylinositol-3 kinase; RAD001: everolimus/Afinitor; RAPTOR: regulatory associated protein of mTOR; RICTOR: RAPTOR independent rapamycin insensitive companion of mTOR; sFCS: charcoal stripped FCS; SGK1: serum and glucocorticoid regulated kinase 1, rapalogue rapamycin analogue.

## Competing interests

JMWG, RIN and IRH are in receipt of research funding from AstraZeneca. SMG is an employee of AstraZeneca. NJJ and the work included in this study was predominantly research funded by AstraZeneca. The remaining authors have no conflict of interest.

## Authors’ contributions

As Principal Investigator, JMWG conceived the study, participated in its design and execution and helped to draft and provide critical revision of the manuscript. RIH, RIN and SMG participated in the design of the study and provided further critical revision of the manuscript. NJJ drafted the manuscript, designed the experiments and carried out cell culture, Western blotting, ICC, proliferation, viability assays and PCR and interpreted the data. CMD, HJM and DB designed and carried out growth studies, analysed and interpreted this data. HJM also carried out migration assays. All authors read and approved the final manuscript.
